# Prognostic value of Cranial Ultrasound combined with Serum Neuron-Specific Enolase and Creatine Kinase-MB Isoenzyme Measurement in Neonatal Hypoxic-Ischemic Encephalopathy

**DOI:** 10.12669/pjms.42.7.16208

**Published:** 2026-07

**Authors:** Bin Wang, Shudan Yin, Xiangyang Zhang

**Affiliations:** 1 Bin Wang, Department of Ultrasound, Xian People’s Hospital, Xi’an Fourth Hospital, Xi’an, Shaanxi Province 710199, P. R. China; 2 Shudan Yin, Department of Ultrasound, Xian People’s Hospital, Xi’an Fourth Hospital, Xi’an, Shaanxi Province 710199, P. R. China; 3 Xiangyang Zhang, Department of Ultrasound, Xian People’s Hospital, Xi’an Fourth Hospital, Xi’an, Shaanxi Province 710199, P. R. China

**Keywords:** Cranial ultrasound, Creatine kinase isoenzyme-MB, Neuron-specific enolase, Neonatal hypoxic-ischemic encephalopathy, Prognosis

## Abstract

**Objectives:**

To investigate the prognostic value of cranial ultrasound combined with serum neuron-specific enolase (NSE) and creatine kinase isoenzyme-MB (CK-MB) levels for evaluating clinical outcomes in neonates with hypoxic-ischemic encephalopathy (HIE).

**Methodology:**

A retrospective study was conducted using clinical data from 126 children with HIE admitted to Xi’an Fourth Hospital from November 2024 to December 2025 (study group). Clinical data from 126 healthy newborns were used as the control group. All the subjects underwent cranial ultrasound and NSE and CK-MB detection. Cranial ultrasound parameters and NSE and CK-MB levels were recorded, correlations among the indicators were analyzed, and the value of each indicator for predicting HIE prognosis was evaluated.

**Results:**

The peak systolic velocity (Vs) (t=18.508; P<0.001) and peak diastolic velocity (Vd) (t=22.741; P<0.001) were lower in the study group than in the control group, whereas the cerebral parenchymal echo intensity (t=26.068; P<0.001), lateral ventricle width (t=34.343; P<0.001), NSE level (t=35.618; P<0.001), and CK-MB level (t=30.276; P<0.001) were greater. Poor prognosis was associated with significantly lower Vs and Vd and greater cerebral parenchymal echo intensity, lateral ventricle width, and NSE and CK-MB levels (P<0.001). NSE and CK-MB levels in the study group were negatively correlated with Vs (r=-0.625, P<0.001; r=-0.483, P<0.001) and Vd (r=-0.568, P<0.001; r=-0.516, P<0.001) and positively correlated with cerebral parenchymal echo intensity (r=0.602, P<0.001; r=0.459, P<0.001) and lateral ventricle width (r=0.535, P<0.001; r=0.528, P<0.001). Combined detection showed the highest sensitivity and accuracy for predicting poor prognosis among the evaluated diagnostic methods.

**Conclusion:**

The combination of cranial ultrasound parameters and serum NSE and CK-MB levels is a potential prognostic indicator of HIE.

## INTRODUCTION

Neonatal hypoxic-ischemic encephalopathy (HIE) is a severe complication of the central nervous system (CNS) caused by perinatal asphyxia. It is a major cause of neonatal death and long-term neurodevelopmental disorders.^1^,^2^ Clinical studies have shown that without timely and effective prognostic evaluation and intervention, approximately 20%~30% of children with HIE will develop permanent neurological impairments such as cerebral palsy, mental retardation, and epilepsy.^3^–^5^ Therefore, early and accurate assessment of prognosis in children with HIE is highly important for screening high-risk cases and formulating individualized intervention strategies.

Currently, clinical methods for evaluating the prognosis of HIE primarily include imaging and serum marker testing.^6^ As the preferred imaging method for neonatal craniocerebral examination, cranial ultrasound is noninvasive, convenient, bedside-operable, and repeatable and can clearly reveal characteristic HIE lesions such as cerebral edema, parenchymal injury, and periventricular leukomalacia. However, quantifying the extent of neuronal damage using ultrasound alone is challenging.^7^,^8^ Serum neuron-specific enolase (NSE), which is expressed mainly in neurons and neuroendocrine cells, is released after brain injury and is correlated positively with the severity of brain injury.^9^ A recent study suggested that combining serum miR-199a, S100B, and NSE levels is highly accurate for the diagnosis of neonatal HIE.^10^ On the other hand, myocardial dysfunction, as indicated by elevated serum levels of troponin-T, creatine kinase-MB (CK-MB), and CK-T, is associated with the severity of HIE and mortality.^11^ Among those cardiac markers, CK-MB levels in umbilical cord blood were found to be increased in neonates with HIE. High CK-MB levels are negatively correlated with nervous system functions.^12^ Hence, an increase in serum levels of CK-MB as a result of cerebral ischemia and hypoxia induced by HIE might be an auxiliary indicator for evaluating brain injury.^13^ Those studies suggest that NSE and CK-MB, both as conventional and low-cost examinations, have great potential as biomarkers in HIE. Considering their different characteristics (NSE can better reflect neuronal damage, and CK-MB can reflect HIE-related myocardial injury/systemic hypoxia injury), the diagnostic accuracy of a combination of NSE and CK-MB in HIE would be interesting to explore.

Early identification of HIE is highly important for clinical management. Compared with a single clinical parameter or biomarker, a multi-index predictive strategy has shown higher specificity and sensitivity in distinguishing HIE.^14^ In this study, we hypothesized that combining cranial ultrasound parameters and serum NSE and CK-MB levels might be a potential strategy with high accuracy and reliability in the prognostic assessment of HIE. This study aimed to evaluate the prognostic value of cranial ultrasound combined with serum NSE and CK-MB measurements in children with HIE.

## METHODOLOGY

This retrospective analysis included clinical data from 126 children with HIE at Xi’an Fourth Hospital from November 2024 to December 2025 (study group) and 126 healthy neonates born at our hospital during the same period (control group). HIE was diagnosed on the basis of a previous study: 1) evidence of an acute peripartum or intrapartum event, 2) a pH ≤ 7.00 or base excess ≤ -16 mmol/L within the first hour after birth, 3) an Apgar score < 5 at 3–10 minutes or an ongoing need for resuscitation, and 4) clinical assessment indicating moderate or severe encephalopathy.^15^

### Ethics approval:

Reference KJLL-Z-K-2026032, Date: February 10^th^, 2026.

### Inclusion criteria:

- The study group met the diagnostic criteria for HIE.^15^
- Gestational age 37–42 weeks.
- Complete clinical data.
- The control group had no history of intrauterine distress or birth asphyxia or neurological diseases or other organ injuries.
- Physical examination and Neonatal Behavioral Neurological Assessment (NBNA).

### Exclusion criteria:

- It is complicated by other brain diseases, such as congenital nervous system malformation, genetic metabolic disease, intracranial hemorrhage, and CNS infection.
- Multiple organ dysfunction, such as severe cardiopulmonary disease, liver and kidney failure, and severe infection, can occur.
- Birth weight < 2500 g or > 4000 g.
- Unqualified test samples or poor-quality cranial ultrasound images that could not be interpreted.
- Mothers with severe infection.

### Biochemical and ultrasonographic evaluation procedures:

All patients in both groups underwent cranial ultrasound and serum tests for NSE and CK-MB. To assess serum NSE and CK-MB levels, 3 mL of femoral venous blood was collected within 6–24 hours after birth, placed in vacuum blood tubes without anticoagulant, allowed to stand at room temperature for 30 minutes, and then centrifuged at 3500rmp for 10 minutes. The upper serum layer was separated and stored at -80°C in an ultralow-temperature freezer until analysis. A Mindray BS-800 M fully automatic biochemical analyzer (Shenzhen Mindray Biomedical Electronics Co., Ltd.) was used for analysis. CK-MB levels were measured using the immunoturbidimetric method with a creatine kinase isoenzyme MB assay kit (Shanghai Kehua Bioengineering Co., Ltd.), and NSE levels were measured by chemiluminescence immunoassay using a neuron-specific enolase assay kit (Shandong Zhongxiu Technology Co., Ltd.). All procedures were performed in strict accordance with the instructions of the instruments and corresponding kits. Tests were completed in a double-blind manner by two senior laboratory technicians, and internal quality control was conducted simultaneously to ensure accurate and reliable results.

Cranial ultrasound was performed for both groups within 6–24 hours after birth using a color Doppler diagnostic system equipped with a dedicated high-frequency linear-array neonatal cranial probe, with the frequency set at 7.5–10.0 MHz. Infants were placed in the supine position. Coronal and sagittal sections were scanned through the anterior fontanelle. In addition to qualitative indicators, including cerebral parenchymal echo, ventricular morphology, and sulcal gyrus structure, the following quantitative parameters were recorded:

- Hemodynamic parameters of the middle cerebral artery: peak systolic velocity (Vs) and end-diastolic velocity (Vd);
- Cerebral parenchymal echo intensity was graded from one to four (scores 1–4) using an ultrasound echo intensity scoring system with reference to the thalamic echo;
- Lateral ventricle width: The maximum width of the bilateral lateral ventricular bodies was measured, and the average value was recorded. Examinations were completed in a double-blind manner by two physicians with more than five years of experience in neonatal cranial ultrasound. In cases of disagreement, the final decision was made after departmental consultation.

### Collected indicators:

The following parameters were collected from all patients:

- Cranial ultrasound parameters, including peak systolic velocity (Vs), end-diastolic velocity (Vd), cerebral parenchymal echo intensity, and lateral ventricle width.
- Serum levels of NSE and CK-MB in the two groups.
- Cranial ultrasound parameters and serum levels of NSE and CK-MB in the study group with different prognoses.
- Correlations between serum NSE levels, CK-MB levels, and cranial ultrasound parameters in the study group.
- Predictive value of cranial ultrasound parameters, NSE, and CK-MB for poor prognosis of HIE, including sensitivity, specificity, and accuracy.

### Prognostic Evaluation Criteria:

A total of 126 children with HIE in the study group were followed up for six months to assess long-term prognosis. The follow-up methods included outpatient reexamination, telephone follow-up, and home visits. On the basis of neurodevelopment, physical examination, and imaging reexamination results, the prognosis was classified as good or poor. A good prognosis was defined as follows: normal neurodevelopment; no neurological deficits such as cerebral palsy, intellectual disability, epilepsy, or language disorder; and an NBNA score ≥ 35. Poor prognosis: permanent neurological impairment, including cerebral palsy, mental retardation, epilepsy, motor dysfunction or language delay, and a NBNA score < 35.

### Statistical analysis:

SPSS 26.0 software was used for data analysis. Measurement data are expressed as the mean ± standard deviation (±s), and an independent-sample t test was used for comparisons between groups. Enumeration data are expressed as rates (%), and the χ² test was used for comparisons between groups. The Pearson correlation coefficient was used to analyze the correlation between serum NSE and CK-MB levels and cranial ultrasound parameters. P < 0.05 indicated a statistically significant difference.

## RESULTS

The study group included 126 neonates (70 males and 56 females), with an average gestational age of 37–42 (39.25 ± 1.08) weeks and an average birth weight of 2500–4000 (3265.47 ± 312.58). There were no significant differences in the general characteristics of the two groups (P > 0.05). Table-I,

**Table-I:** Comparison of general data between the two groups.

Group	No. of Cases	Gestational Age (weeks, *x̄*±*s*)	Birth Weight (g, *x̄*±*s*)	Gender (Male/Female)	Underlying Diseases [n (%)]		
					History of gestational hypertension	Premature rupture of membranes	Nuchal cord
Study Group	126	39.25±1.08	3265.47±312.58	70/56	12(9.52)	26(20.63)	19(15.08)
Control Group	126	39.18±1.12	3280.63±305.42	68/58	10(7.94)	24(19.05)	17(13.49)
*t/χ² value*		0.505	0.389	0.064	0.199	0.100	0.130
*P value*		0.614	0.698	0.800	0.655	0.752	0.719

Compared with those in the control group, the Vs and Vd in the study group were significantly lower, whereas the cerebral parenchymal echo intensity, lateral ventricle width, and serum NSE and CK-MB levels were considerably greater (P < 0.05; Table-II)., Of the 126 children with HIE in the study group, 78 had a good prognosis, and 48 had a poor prognosis. Table-III The Vs and Vd values in children with poor prognosis were lower than those in children with good prognosis, while the cerebral parenchymal echo intensity, lateral ventricle width, and serum levels of NSE and CK-MB were greater (P < 0.05).

**Table-II:** Comparison of cranial ultrasound parameters and NSE and CK-MB levels between the two groups (*x̄*±*s*).

Group	n	Vs(cm/s)	Vd(cm/s)	Cerebral parenchymal echo intensity (score)	Lateral ventricle width (mm)	NSE(ng/mL)	CK-MB(U/L)
Study group	126	42.35±6.82	10.26±2.15	2.89±0.72	6.85±1.02	28.64±5.37	30.58±6.12
Control group	126	58.69±7.19	18.53±3.47	1.12±0.25	3.26±0.58	10.25±2.18	11.89±3.25
*t value*		18.508	22.741	26.068	34.343	35.618	30.276
*P value*		<0.001	<0.001	<0.001	<0.001	<0.001	<0.001

**Table-III:** Comparison of cranial ultrasound parameters, NSE and CK-MB in HIE children with different prognoses in the study group (*x̄*±*s*).

Group	n	Vs(cm/s)	Vd(cm/s)	Cerebral parenchymal echo intensity (score)	Lateral ventricle width (mm)	NSE(ng/mL)	CK-MB(U/L)
Good prognosis	78	48.62±8.15	13.57±2.82	2.15±0.58	5.36±0.72	21.35±3.26	23.64±4.58
Poor prognosis	48	32.18±5.67	5.89±1.65	3.92±0.81	8.98±1.11	37.89±6.12	39.15±8.37
*t* value		12.260	17.134	14.263	22.217	19.771	13.439
*P* value		<0.001	<0.001	<0.001	<0.001	<0.001	<0.001

Correlation analysis revealed that serum NSE and CK-MB levels in the study group were significantly negatively correlated with Vs and Vd and significantly positively correlated with cerebral parenchymal echo intensity and lateral ventricle width (P < 0.05; Table-IV). Among the evaluated diagnostic methods, combined detection showed the highest sensitivity and accuracy for predicting poor prognosis in children with HIE. Overall comparisons showed significant differences in sensitivity and accuracy among the diagnostic methods, whereas specificity did not differ significantly (Table-V).

**Table-IV:** Correlations between NSE, CK-MB and cranial ultrasound parameters.

Indicator		Vs	Vd	Cerebral parenchymal echo intensity	Lateral ventricle width (mm)
NSE	r value	-0.625	-0.568	0.602	0.535
	*P* value	<0.001	<0.001	<0.001	<0.001
CK-MB	r value	-0.483	-0.516	0.459	0.528
	*P* value	<0.001	<0.001	<0.001	<0.001

**Table-V:** Analysis of the predictive value of the cranial ultrasound parameters NSE and CK-MB for poor prognosis in HIE patients.

Diagnostic method	Sensitivity	Specificity	Accuracy
Vs	72.92%(35/48)	85.90%(67/78)	80.95%(102/126)
Vd	70.83%(34/48)	84.62%(66/78)	79.37%(100/126)
Cerebral parenchymal echo intensity	77.08%(37/48)	85.90%(67/78)	82.54%(104/126)
Lateral ventricle width	75.00%(36/48)	86.54%(68/78)	82.54%(104/126)
NSE	79.17%(38/48)	86.54%(68/78)	84.13%(106/126)
CK-MB	77.08%(37/48)	85.90%(67/78)	82.54%(104/126)
Combined detection	97.92%(47/48)	89.74%(70/78)	92.86%(117/126)
*χ²* value	13.788	1.075	26.507
*P* value	0.032	0.983	<0.001

## DISCUSSION

HIE is a leading cause of neonatal death and long-term neurodevelopmental disorders.^16^ The incidence of long-term neurological sequelae in children with moderate to severe HIE can reach as high as 30–50%.^17^ Early diagnosis and accurate prognostic evaluation are important for timely interventions and improving outcomes.^18^ In this study, 126 children with HIE were included, and the results revealed that the cranial ultrasound parameters NSE and CK-MB have strong prognostic value for HIE. Serum indicators and cranial ultrasound parameters are significantly correlated, and the combined strategy can improve the sensitivity and accuracy of predicting poor prognosis in HIE patients.

NSE and CK-MB are two conventional and low-cost examinations in clinical. The core innovation of this study lies in the simultaneous integration of cranial ultrasound hemodynamics, morphological quantitative indicators, and serum NSE and CK-MB dual markers for the first time; the goal is to construct a multidimensional joint evaluation system, fill the limitations of single imaging or single serological indicators in evaluating HIE prognosis, and provide a new evidence-based basis for the stratified management of neonatal HIE prognosis. This key academic information was added to the medical literature in the field of neonatal brain injury in this study. The results of this study revealed that HIE was associated with significantly lower Vs and Vd of the middle cerebral artery. Moreover, cerebral parenchymal echo intensity, lateral ventricle width, and serum NSE and CK-MB levels were significantly greater (P < 0.05) in the HIE group than in the control group, which is consistent with the findings of previous studies.

A report by Fox A et al.^19^ revealed that compared with those in healthy newborns, the Vs and Vd of the middle cerebral artery in children with HIE were reduced by approximately 30%, and the lateral ventricle width was doubled. It was suggested that cerebral ischemia and hypoxia caused by perinatal asphyxia lead to cerebral vasospasm and insufficient cerebral perfusion, resulting in abnormal hemodynamic parameters, whereas cerebral edema and parenchymal injury cause increased parenchymal echodensity and ventricular dilatation.^19^

Studies by Lv H et al.^6^ and Wan B et al.^12^ also confirmed that serum levels of NSE and CK-MB within 24 hours after birth were significantly elevated in children with HIE and positively correlated with the severity of asphyxia, which is consistent with the findings of this study. It is plausible that ischemia and hypoxia disrupt the integrity of neuronal cell membranes, resulting in the massive release of intracellular NSE and brain--distributed CK-MB into the blood, making them important molecular markers of brain injury.^20^–^22^ In contrast to previous studies, this study simultaneously included quantitative cranial ultrasound parameters and serum biomarkers, thereby verifying the association between these indicators and HIE pathogenesis from both a morphological and molecular perspective and providing a more comprehensive laboratory basis for early screening of HIE.

In the study group, the Vs and Vd values were significantly lower in children with poor prognosis than in those with good prognosis, whereas the cerebral parenchymal echo intensity, lateral ventricle width, and serum levels of NSE and CK-MB were significantly greater (P < 0.05). These results reveal a direct relationship between the severity of abnormal indicators and HIE prognosis, which is consistent with the findings of previous studies. Lee DA et al.^23^ reported that a greater percentage of children with poor prognosis had serum NSE concentrations > 35 ng/mL, and those whose lateral ventricle width was > 8 mm on cranial ultrasound had a greater incidence of long-term cerebral palsy.

This study further quantified the differences in each indicator. The results revealed that the Vs and Vd in the poor prognosis group were approximately 30% lower, the cerebral parenchymal echo intensity was approximately 80% higher, and the serum NSE and CK-MB levels were approximately 90% and 70% higher, respectively, than those in the good prognosis group. These data more directly reflect the positive correlation between the degree of abnormal indicators and the severity of brain injury. Compared with previous studies in which only blood flow parameters or serum markers were detected, this study further quantified the amplitude of abnormal indicators, clarified the quantitative correlation between the degree of abnormal indicators and the degree of brain injury, and provided a quantifiable reference standard for rapid clinical judgment of the degree of HIE lesions. These quantitative analysis results are additional clinical data that have not been fully reported in previous similar studies and have direct clinical application value.

The present study also revealed significant correlations between serum NSE and CK-MB levels and cranial ultrasound parameters; these parameters were negatively correlated with Vs and Vd and positively correlated with cerebral parenchymal echo intensity and lateral ventricle width (P < 0.05). This correlation was also confirmed in a study by Huang HZ et al.^24^, which revealed that serum NSE levels were closely associated with the blood flow status of the middle cerebral artery in HIE infants. These findings suggest that the severity of cerebral ischemia–hypoxia is correlated with the degree of cerebral hypoperfusion and hemodynamic disturbance. Moreover, progressive neuronal injury increases serum biomarker levels, indicating a concordant relationship between ultrasonographic parameters and biochemical markers of brain injury.^24^

In contrast, the current study incorporated morphological indicators such as cerebral parenchymal echo intensity and lateral ventricle width, demonstrating a correlation between serum biomarkers and structural brain changes and providing integrated insight into brain injury at both the molecular and morphological levels. In addition, the correlation coefficients of serum NSE with all cranial ultrasound parameters were greater than those of CK-MB, suggesting that NSE is more specific in reflecting the degree of brain injury. This finding is consistent with tissue-specific characteristics: NSE is expressed mainly in neurons, whereas CK-MB is also abundantly expressed in myocardial tissue,^25^,^26^ providing a reference for the clinical selection of core evaluation indicators.

The core finding of this study is that the combination of cranial ultrasound parameters with serum NSE and CK-MB showed the highest sensitivity and accuracy among the evaluated diagnostic methods for predicting poor prognosis in infants with HIE, suggesting the potential clinical value of combined detection and being consistent with the trend of combined evaluation research in recent years. Building on previous studies, this study included CK-MB and quantitative parameters of cranial ultrasound hemodynamics and morphology, making the combined detection system more comprehensive and potentially improving its predictive efficiency. Compared with traditional single-indicator detection, combined detection may offer three advantages.

First, cranial ultrasound is used to intuitively reflect changes in brain structure and hemodynamics and clarify the location and severity of brain injury. Second, serum NSE and CK-MB levels accurately reflect molecular changes associated with neuronal injury, improving the sensitivity of early diagnosis. Third, multi-indicator complementarity reduces missed and misdiagnosed cases caused by single indicators. In addition, the specificity of each indicator was similar (84.62–86.54%), with no significant difference between groups (P > 0.05). The combined detection method integrated multi-indicator information and slightly increased the specificity to 89.74%, further improving the prediction reliability.

### Limitations:

This was a single-center retrospective study with a relatively small sample size. The optimal cutoff values for ultrasound parameters, NSE, and CK-MB for predicting the clinical outcomes of HIE were not confirmed. Additional multicenter, large-sample prospective studies are needed to further validate the clinical value of combined detection and to explore the optimal cutoff value for each indicator, thereby providing a more accurate reference standard for clinical diagnosis and treatment. Additionally, neurological outcomes were assessed only at six months, which does not adequately capture long-term sequelae such as cognitive delay, cerebral palsy, or epilepsy that may develop later in childhood. The joint detection scheme constructed in this study has significant advantages, such as noninvasiveness, convenience, bedside operability, and low cost, and is especially suitable for rapid bedside assessment in primary medical institutions and neonatal intensive care units. It can be widely promoted and applied to the early prognosis screening of children with HIE. This is the core clinical advantage that distinguishes this study from high-end imaging examinations and invasive detection methods, greatly enhancing the clinical translation value and promotion significance of the research results.

## CONCLUSIONS

To summarize, this study suggested that combining cranial ultrasound parameters, NSE, and CK-MB is a useful tool in predicting poor prognosis in HIE patients and may be useful for providing appropriate CNS and cardiovascular support in addition to managing the primary condition in infants with HIE.

### Recommendations:

Based on the findings and shortcomings of this study, future in-depth research in the following directions is needed:

- Conducting multicenter, large-sample prospective cohort studies to expand the representativeness of the study population and verify the universality and stability of this joint detection system;
- Determining the optimal critical values of cranial ultrasound parameters, NSE, and CK-MB for predicting the prognosis of HIE through statistical methods and establishing a standardized clinical testing and interpretation process;
- Extending the follow-up period, observing the long-term neurological development outcomes of children, and further verifying the predictive value of the joint evaluation system for long-term sequelae such as cerebral palsy and intellectual disability;
- Combining artificial intelligence algorithms to construct a prognostic prediction model, further improving the accuracy and automation level of evaluation and promoting the intelligent development of HIE prognostic assessment.

### Author contributions:

**BW:** Literature search, study design and manuscript writing.

**SY and XZ:** Data collection, data analysis and interpretation. Critical review.

**BW:** Manuscript revision and validation and is responsible for the integrity of the study.

All the authors have read and approved the final manuscript.
